# Validation of Expression Patterns for Nine miRNAs in 204 Lymph-Node Negative Breast Cancers

**DOI:** 10.1371/journal.pone.0048692

**Published:** 2012-11-07

**Authors:** Kristin Jonsdottir, Susanne R. Janssen, Fabiana C. Da Rosa, Einar Gudlaugsson, Ivar Skaland, Jan P. A. Baak, Emiel A. M. Janssen

**Affiliations:** 1 Department of Pathology, Stavanger University Hospital, Stavanger, Norway; 2 Free University, Amsterdam, The Netherlands; H.Lee Moffitt Cancer Center & Research Institute, United States of America

## Abstract

**Introduction:**

Although lymph node negative (LN-) breast cancer patients have a good 10-years survival (∼85%), most of them still receive adjuvant therapy, while only some benefit from this. More accurate prognostication of LN- breast cancer patient may reduce over- and under-treatment. Until now proliferation is the strongest prognostic factor for LN- breast cancer patients. The small molecule microRNA (miRNA) has opened a new window for prognostic markers, therapeutic targets and/or therapeutic components. Previously it has been shown that miR-18a/b, miR-25, miR-29c and miR-106b correlate to high proliferation.

**Methods:**

The current study validates nine miRNAs (miR-18a/b miR-25, miR-29c, miR-106b, miR375, miR-424, miR-505 and let-7b) significantly correlated with established prognostic breast cancer biomarkers. Total RNA was isolated from 204 formaldehyde-fixed paraffin embedded (FFPE) LN- breast cancers and analyzed with quantitative real-time Polymerase Chain Reaction (qPCR). Independent T-test was used to detect significant correlation between miRNA expression level and the different clinicopathological features for breast cancer.

**Results:**

Strong and significant associations were observed for high expression of miR-18a/b, miR-106b, miR-25 and miR-505 to high proliferation, oestrogen receptor negativity and cytokeratin 5/6 positivity. High expression of let-7b, miR-29c and miR-375 was detected in more differentiated tumours. Kaplan-Meier survival analysis showed that patients with high miR-106b expression had an 81% survival rate vs. 95% (P = 0.004) for patients with low expression.

**Conclusion:**

High expression of miR-18a/b are strongly associated with basal-like breast cancer features, while miR-106b can identify a group with higher risk for developing distant metastases in the subgroup of Her2 negatives. Furthermore miR-106b can identify a group of patients with 100% survival within the otherwise considered high risk group of patients with high proliferation. Using miR-106b as a biomarker in conjunction to mitotic activity index could thereby possibly save 18% of the patients with high proliferation from overtreatment.

## Introduction

For breast cancer in general lymph node status is still the strongest prognostic factor. Lymph Node negative (LN-) breast cancer patients constitute ∼60% of all breast cancer cases. Nowadays almost all of them receive adjuvant treatment despite the fact that ∼75% have a good 10-years survival. Over the past decade, especially lymph node negative breast cancer has been intensively investigated by array-based gene expression profiling. These studies have identified new subtypes like luminal A and -B, basal-like and Her2/neu-overexpressing cancers that correlate with different survival outcome [1], [2]. Luminal type constitutes the biggest group of breast cancers and is associated with oestrogen receptor alpha (ERα) positivity which can be further subdivided into luminal A with low and luminal B with high proliferation. Approximately 7–12% of all breast cancers have a Her2 amplification and form the Her2/neu-overexpressing subtype. In contrast, basal-like breast cancers express no hormonal receptors, are cytokeratin 5/6 or 14 positive, associated with poor prognosis and constitute 14–20% of all breast cancer cases [3]–[5]. Specific gene signatures have also been found to predict therapy response or resistance, hormone receptor status and for identification of patients at risk of distant recurrence following surgery.

In the majority of the prognostic signatures, proliferation associated genes are strongly represented. This confirms numerous studies which have shown that proliferation is the strongest prognostic factor in lymph node negative breast cancer, either measured by mitotic activity index (MAI), Ki67 or phosphohistone H3 (PPH3) [6]–[8]. In comparison with several prognostic signatures, single genes related to proliferation had similar or even better prognostic value [9]. Although gene expression signatures and proliferation can add prognostic value to the established prognostic markers for LN- breast cancer, still many breast cancer patients are under- and over-treated. Therefore a better understanding of the complex biology of the different breast cancer subtypes is required.

miRNAs have recently been described as a new mechanism for posttranscriptional regulation of protein transcription. miRNAs are a group of small non-coding RNAs (19–25 nt) that regulate the degradation (perfect match) and translation (imperfect match) of their target mRNA. One miRNA can target up to 200 different mRNAs, tend to be pathway specific [10]–[12] and are involved in a wide range of biological and pathological processes [13]. In contrast to mRNA, miRNAs are much more stable in formalin fixed paraffin embedded tissue [14] which makes them easy to investigate and increase their potential use as biomarkers in routine diagnostics for breast cancer.

In a previous publication we investigated miRNA expression by microarray analysis in total RNA isolated from fresh frozen tumour from 104 LN- breast cancers, and showed amongst others that miR-106b, miR-18a/b, miR-25, miR-29c and miR-505 were strongly correlated to high proliferation [15] while others correlated with both ERα negativity and CK5/6 positivity. As with all microarray data, these previously published data need to be validated by other methods and in an independent study cohort. In order to achieve this, nine selected microRNAs (let-7b, miR-18a, miR-18b, miR-106b, miR-25, miR-29c, miR-375, miR-424, miR-505) were tested by qPCR in total RNA isolated from 204 new LN- breast cancers patients under the age of 71 and investigated correlations with classic clinicopathological features like proliferation (measured by MAI, PPH3 and Ki67), ERα, progesteron receptor (PR), Her2, and CK5/6.

## Materials and Methods

### Patients and Pathology

The study was approved by the Regional Ethics Committee before the study started. All 240 patients were diagnosed with first onset invasive operable (T_1,2_N_0_M_0_) breast cancer at the Stavanger University Hospital, between January 1, 1996 and December 31, 1998. The following samples were excluded; 34 cases without enough tumour material, one sample had bad fixation and one had too much inflammation, leaving 204 patients for analysis. There were no significant differences between the original 240 and final 204 cases in any of the clinicopathologic features analyzed. The patients were all treated according to the national guidelines of the Norwegian Breast Cancer Group at that time. The auxiliary fat was macroscopically examined and all detectable lymph nodes (median: 12, range: 4–27) were prepared for histology. All tissues were cut in 5 millimeter thick slices, fixed in buffered 4% formaldehyde and embedded in paraffin. Four micrometer histological sections were made and stained with haematoxylin-erythrosin-safran (HES). Histological type and grade were assessed by two pathologists (EG, JB) with considerable experience in breast pathology, according to the World Health Organization criteria [16]. Grade was carefully assessed according to the Nottingham modification [17], [18]. The MAI was assessed as described elsewhere [19].

**Figure 1:**
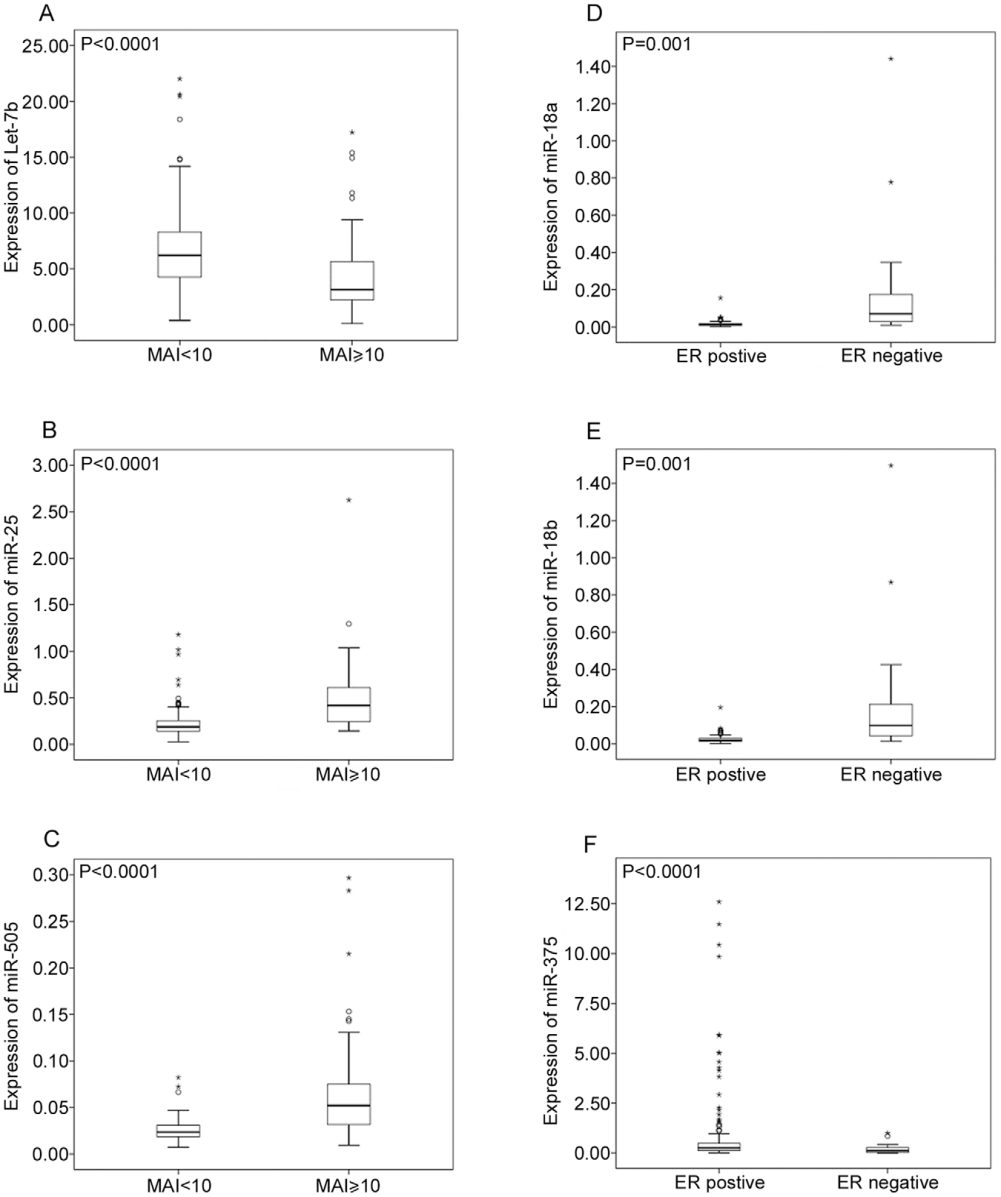
Expression level of miRNAs for different prognostic features. Independent T-test was used to determined significant relationship. Let-7b is down-regulated in high MAI ≥10 (A), miR-25 (B) and miR-505 (C) are expressed higher in MAI ≥10. MiR-18a (D) and miR-18b (E) are expressed higher in ERα negative patients, while miR-375 (F) is down-regulated in ERα negative breast cancer patients.

**Table 1:** Independent t-test between miRNAs and different clinical features for breast cancer.

	High Proliferation	ER−	PR−	TNP−	CK5/6+
**Let-7b**	P<0.0001	P<0.0001	P = 0.005	P<0.0001	P<0.0001
miR-106b	P<0.0001	P<0.0001	P = 0.001	P<0.0001	P = 0.044
miR-18a	P<0.0001	P = 0.001	P = 0.001	P = 0.003	P = 0.003
miR-18b	P<0.0001	P = 0.001	P = 0.003	P = 0.002	P = 0.002
miR-25	P<0.0001	P<0.0001	P<0.0001	P<0.0001	P = 0.004
**miR-29c**	P<0.05*	P<0.0001		P<0.0001	P<0.0001
**miR-375**		P<0.0001			P<0.0001
miR-424					
miR-505	P<0.0001	P<0.0001	P = 0.001	P = 0.001	P = 0.002

Proliferation includes MAI, PPH3 and Ki67. Star indicated that Ki67 did not give P-value under 0.05. The miRNAs with underscore/fat are inversely correlated to the different clinical features, meaning that low expression of let-7b is significantly associated to high proliferation.

### Immunohistochemistry

ERα and PR, PPH3, Ki67, CK5/6 and Her2 expression were determined by immunohistochemistry (IHC) in whole sections. Antigen retrieval and IHC techniques were based on DAKO technology as described previously [8].

**Figure 2:**
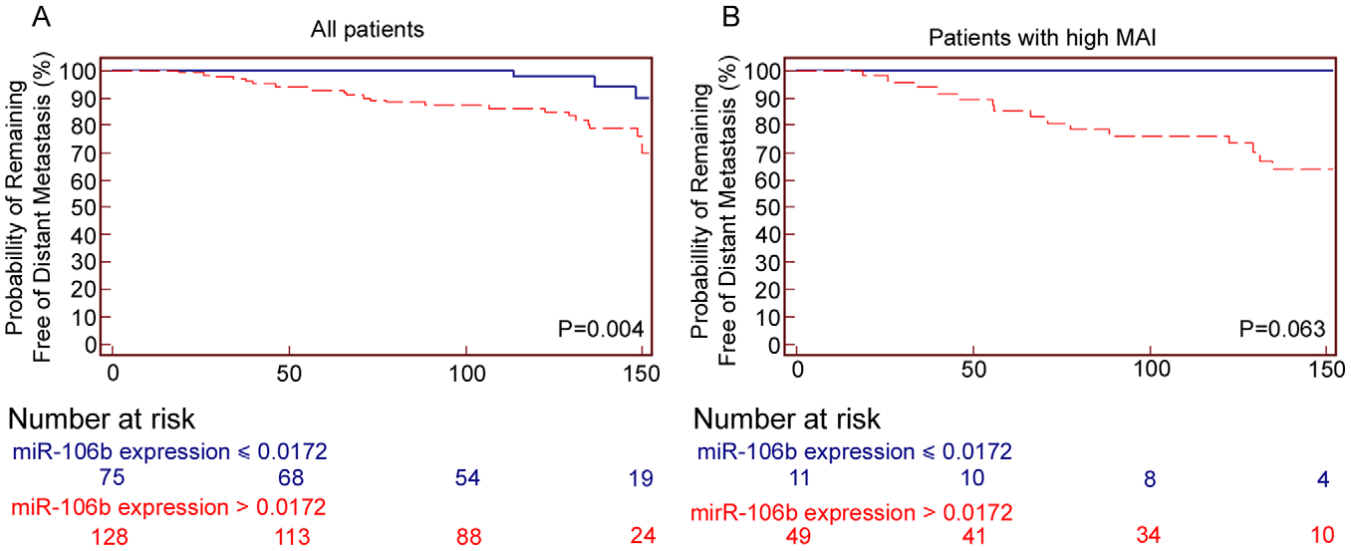
Long-term distant metastasis free survival curves according to miR-106b expression level and miR-106b expression in patients with MAI≥10.

**Table 2:** Distant metastasis free survival in lymph node-negative breast cancer patient with Kaplan Meier and cox multivariate analysis.

Characteristic	Distant metastasis			
	Event/at risk(%)	Log-rankP-value	HR	95% CI
**Age**				
<55 years	13/92 (86)	0.375	1.4	0.7–2.9
≥55 years	16/112 (86)			
**Tumour diameter**				
<2 cm	16/148 (89)	0.045	2.1	1.0–4.4
≥2 cm	13/56 (77)			
**Nottingham** **grade**				
1	2/64 (97)	0.010	5.5	1.3–23.2
2 and 3	27/140 (81)			
**ER**				
Positive ≥1%	20/164 (88)	0.335	1.5	0.7–3.4
Negative	8/39 (80)			
**PR**				
Positive ≥10%	17/123 (86)	0.798	1.1	0.5–2.3
Negative 0–9%	12/81 (81)			
**Her2**				
0–1+	20/176 (89)	0.002	3.2	1.5–7.1
2+–3+	9/25 (64)			
**MAI**				
<10	14/144 (90)	0.006	2.7	1.3–5.8
≥10	15/60 (75)			
**MAI**				
MAI 0–2	6/100 (94)	0.004	3.2	1.1–9.3
MAI 3–9	8/44 (81)		4.6	1.7–12.0
MAI ≥10	15/60 (75)			
**Ki67**				
0–9%	8/94 (92)	0.018	2.6	1.1–6.1
10–100%	19/88 (78)			
**PPH3**				
<13	9/122 (93)	0.002	3.5	1.5–7.9
≥13	20/80 (75)			
**CK5/6**				
Negative	23/175 (87)	0.407	1.5	0.6–3.6
Positive	6/28 (79)			
**Triple Negative**				
Positive	23/174 (87)	0.362	1.5	0.6–3.7
Negative	6/30 (80)			
**Let-7b**				
>3.2792	14/146 (90)	0.007	2.7	1.3–5.7
≤3.2792	15/58 (74)			
**miR-106b**				
≤0.0172	4/75 (95)	0.004	4.3	1.5–12.6
>0.0172	25/128 (81)			
**miR-18a**				
≤0.0121	6/77 (92)	0.083	2.2	0.9–5.4
>0.0121	22/125 (82)			
**miR-18b**				
≤0.025	10/114 (91)	0.033	2.3	1.0–4.9
>0.025	18/87 (79)			
**miR-25**				
≤0.2509	11/119 (91)	0.022	2.4	1.1–5.3
>0.2509	17/84 (80)			
**miR-29c**				
>1.1674	7/78 (91)	0.113	2.0	1.0–4.3
≤1.1674	22/126 (83)			
**miR-375**				
≤0.3487	14/136 (90)	0.054	2.0	1.0–4.3
>0.3487	14/67 (79)			
**miR-424**				
≤0.7702	18/146 (88)	0.388	1.4	0.7–2.9
>0.7702	11/57 (81)			
**miR505**				
≤0.0182	3/40 (93)	0.295	1.9	0.6–6.2
>0.0182	25/163 (85)			

*HR* hazard ratio, *CI* confidence interval.

### Quantification of PPH3, Basal Cytokeratin, ERα, PR, Ki67 and Her2

The PPH3 index was assessed as described elsewhere [20]. For measuring % of Ki67 positive cells we used the semi-automatic interactive computerized QPRODIT system (Leica, Cambridge), as described in [21]. The percentage of CK5/6 positive tumour cells in each cancer was scored using a continuous scale of 0–100%. In the final analysis all tumours with any CK5/6 staining in tumour cells were grouped as being positive as described before [22]. ERα was scored positive if ≥1% of tumours cells showed nuclear staining and all others were scored negative. PR was scored as positive when nuclear staining was present in >10%, and scored negative when <10% of the of the tumour cells had nuclear staining. Her2 was scored according to the DAKO Hercep Test scoring protocol. All 2+ and 3+ cases were regarded as positive. All sections were independently scored by two pathologists. Triple negative breast cancers (TNP) are defined by negativity for ERα (0%), PR (<10%) and Her2 (0 and 1+).

**Table 3:** Kaplan-Meier survival analysis for Her2 and miR-106b.

Characteristic	Distant metastases	
	Event/at risk (%)	Log-rank P-value
**Her2 negative**		
miR-106b ≤0.0172	3/69 (96)	0.015
miR-106b >0.0172	17/107 (84)	
**Her2 positive**		
miR-106b ≤0.0172	1/6 (83)	0.155
miR-106b >0.0172	8/18 (56)	

### RNA-isolation and qPCR for FFPE Tissue

All sections were evaluated by an experienced breast pathologist (EG) who selected an area with at least 50% tumour cells for RNA isolation. Five sections of 10 µm were used for RNA isolation. Tumour tissue was isolated by macrodissection from the slides. Total RNA was isolated using miRNeasy for FFPE kit (Qiagen, Valencia, CA) according to the protocol provided by the manufacturer. All samples were analyzed on a Nanodrop instrument (Thermo Fisher Scientific, Waltham, USA). cDNA samples were made out 20 ng of total RNA using the Universal cDNA synthesis kit (Exiqon A/S, Vedbaek, Denmark) according to the manufacturer’s recommendations. A 8 µl volume of 80x dilution of cDNA was used in each of the real-time PCR reactions with SYBR® green master mix and miRNA LNA™ PCR primer sets (both from Exiqon A/S), following the manufacturer’s instructions. All samples were run in triplicate. In order to find candidates that could be used as control genes, we used the previously published miRNA array data [15] and the free programs Normfinder [23] and GeNorm [24]. miR-24 and miR-26b showed the least variation between the 104 samples. U6 was also included as a reference gene. The average Ct-values of the triplicates for hsa-miR-24 (mean Ct = 22.73, std. = 1.33) and hsa-miR-26b (mean Ct = 26.17, std. = 1.66) were used as the endogenous reference and relative expression of target genes was calculated via the equation 2^−ΔCt^. U6 was later excluded by GeNorm and Normfinder because of too much variation (mean Ct = 26.43, std. 1.87) between the different patient samples.

**Figure 3:**
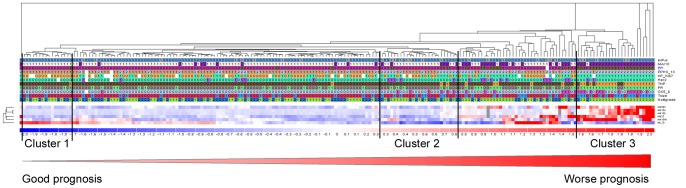
Supervised hierarchical clustering for ERα. Genes were filtered using analysis of variance, p-value ≤0.0001 and absolute correlation for ERα. The heat-map diagram shows the result of the two-way hierarchical clustering of miRNAs and samples. Good prognosis indicates patients with DMFS, while worse prognosis refers to patients with distant metastasis or who have died of distant metastasis. Each row represents a miRNA and each column represents a patient sample. The miRNA clustering tree is shown on the left, and the sample clustering tree appears at the top. The colour scale shown at the bottom illustrates the relative expression level of a miRNA across all samples: red colour represents an expression level above mean, blue colour represents expression lower than the mean. Gray colour means that the specific miRNA has not been successfully detected with qPCR. Numbers for clinicopathological features indicate the following: EOFUS (0 = no distant metastasis, 1 = distant metastasis), MAI10 (0<10, 1≥10), ERα (0<1% positive tumour cells, 1≥1% positive tumour cells), PPH3_13 (0<13, 1≥13), KP_Ki67 (0<10%, 1≥10%), Her2 (0 = 0 or 1+, 1 = 2+ or 3+), TNP (0 = positive for either ERα/PR/Her2, 1 = negative for ERα and PR and Her2), PR (0≤10% positive tumour cells, 1>10% positive tumour cells), CK5/6 (0 = no staining, 1 = any percentage of positive tumour cells), Tsize (Tumour size: 0≤2cm, 1>2 cm) and Nottgrade (Nottingham grade: 1 = grade 1, 2 = grade 2, 3 = grade 3).

### Survival Endpoints

For survival analysis, the end point used was distant metastasis free survival (DMFS) and which we defined as any recurrence at a distant site. Patients were censored from the date of the last follow-up visit for death from causes other than breast cancer, local or regional recurrences, and the development of a second primary cancer, including contra lateral breast cancer. If a patient's status during follow-up indicated a confirmed metastasis without a recurrence date, the follow-up visit date was used. Age, time to first recurrence and survival time were calculated relative to the primary diagnosis date.

**Figure 4:**
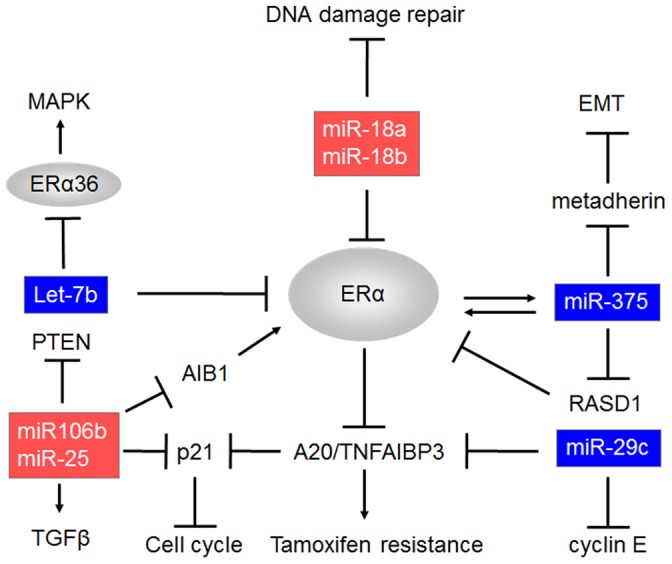
An illustration of the miRNAs impact on ERα, based on literature and results presented in this paper.

### Statistical Analyses

SPSS (SPSS, Chicago, Illinois, USA) for Windows version 18.0 was used. Kaplan–Meier survival curves were constructed and differences between groups were tested by the log rank test. The relative importance of potential prognostic variables was tested using univariate and multivariate (for 196 patients all features were available) Cox-proportional hazard analysis (forward, Wald) and expressed as a Hazards Ratio (HR) with 95% confidence intervals (CI). Dchip software (version 31 March 2009) (www.dchip.org) was used for hierarchical cluster analysis. The qPCR data were standardized as follows, subtracting mean and divided by standard deviation. Genes were filtered using analysis of variance, p-value ≤0.01 and absolute correlation (meaning including genes with opposite profile). These gene lists were used to classify samples by cluster analysis and linear discriminate analysis (LDA). The optimal expression threshold for miR-106b was determined by Receiver Operating Characteristic (ROC) curve analysis (MedCalc statistical software v. 9.3.7, MedCalc, Mariakerke, Belgium.

**Table 4:** Pathway analysis of the nine microRNAs by means of Diana microT 4.0 (beta version), PicTar and Targetscan 5.

microRNA	KEGG pathway	Prediction Pathway	KEGG-pathway ID	Highest -ln(p-value)	Number of Genes	Genes
Let 7b	MAPK signaling pathway	PTTS5 5Diana 4.0	hsa04010	12.3 (PT)	27	MAP4K3, MAP4K4, DUSP4, CSDE1, TGFBR1, PDGFB, MAP3K7IP2, DUSP9, FASLG, CACNA1D, DUSP16, MAP3K3, PAK1, FGF11, PPP3CA, ACVR1B, PLA2G3, NLK, DUSP1, ACVR1C, CASP3
miR-18a	P53-signaling pathwayUbiquitin mediated proteolysis	Diana 4.0+ TSPT + TS5	hsa04115hsa04120	13.0 (Diana 4.0)5.3 (TS5)	55	THBS1, ATM, CDK2, CCND2, IGF1UBE2G1, UBE2Z, MAP3K1, PIAS3, NEDD4
miR-18b	P53-signaling pathway	TS5Diana-4.0	hsa04115	11.9 (Diana 4.0)	5	THBS1, ATM, CDK2, CCND2, IGF1
miR-106b	TGF-beta signaling pathway	PTTS5Diana-4.0	hsa04350	17,2 (PT)	12	E2F5, RBL2, DOCK5, ZFYVE9, SMAD7, SMAD6, RBL1, SMAD5, PPP2CA, ACVR1B, BMPR2, EP300
miR-25	Phosphatidylinositol signaling system	PTTS5Diana-4.0	hsa04070	7.5 (TS5)	8	SYNJ1, ITPR1, CALM1,CALM2,CALM3, IMPA2, PIP5K1C, PIP4K2C, PIP5K3, PIK3R3
miR-29c	ECM-receptor interaction	PTTS5Diana-4.0	hsa04512	47.16 (Diana 4.0)	20	COL6A3, COL4A6, FN1, COL5A1, COL1A1, LAMA2, COL3A1, DAG1, ITGA2, ITGA6, COL4A4, COL5A3, HSPG2, COL5A2, COL1A2, ITGA11, COL2A1, LAMC1, COL4A1, COL11A1
miR-375	Biotin metabolism	PTTS5Diana-4.0	hsa00780	6.36 (TS5)	1	HLCS
miR-424	Prostate cancer	TS5Diana-4.0	hsa05215	13.3(TS5)	15	BCL2, CCNE1, E2F3, CSDE1, IGF1R, MAP2K1, PIK3R1, SOS1, FGFR1, FOXO1, IKBKB, IGF1, AKT3, CCND1, RAF1
miR-505	Tight junction	TS5Diana-4.0	hsa04530	4.07 (TS5)	4	ACTN2, VAPA, JAM3, MYH10

Only the most significant (from the top three) pathway that is common in all three software programmes is mentioned. Abbreviations: Diana 4.0 = Diana microT 4.0 (beta version), PT = PicTar, TS5 = Targetscan 5.

## Results

Median age was 57 (range 30–71 years) with median follow-up of 122 months (range 10–178 months). Twenty-nine patients developed distant metastasis or died from breast cancer. Table 1 shows the significant associations between the nine miRNAs and the clinicopathological features. The most significant differences between the miRNAs and the clinicopathological features are illustrated by boxplots in figure 1 and all other boxplots are shown in figure S1, S2, S3, S4, S5. Three of the nine miRNAs studied (let-7b, miR-29c, and miR-375) are inversely correlated to the classical prognostic features (proliferation, ER/PR and tumour size) and can be regarded as tumour suppressive. Let-7b was the most differently expressed miRNA (std. = 3.99) and low expression of let-7b was significantly linked to high proliferation (Figure 1A), TNP, ERα negative, CK5/6 positivity. miR-29c showed significant (P<0.0001) associations to high proliferation, TNP, ERα negativity and CK5/6 positivity. Low expression of miR-375 was only significantly associated to ERα negativity (P<0.0001, Figure 1F) and CK5/6 positivity. miR-424 was not associated to any of the different clinicopathological features. High expression of miR-25 was significantly associated to ERα negativity, high proliferation (Figure 1B), TNP and CK5/6 positivity. Furthermore miR-25 correlated very strongly to miR-106b (corr. = 0.428, P = 0.0001). miR-505 was also expressed at low levels in all patients. Strong and significant associations between high miR-505 expression and ERα negativity, high proliferation, TNP (Figure 1C) and CK5/6 positivity were found. High expression of miR-18a and miR-18b was also significantly associated with high proliferation, ERα negativity (Figure 1D and 1E), TNP and CK5/6 positive breast cancers. miR-18a and miR-18b were also expressed at lower levels in all samples and their expression profile are strongly correlated to each other (Pearson correlation = 0.995, p<0.0001).

Table 2 shows the survival and hazard ratios for the classical tumour characteristics and the nine miRNAs for DMFS. The cut-off values used for the survival analyses for each microRNA were obtained by ROC analysis (Table S1 and Figure S6). Median and 3-quantiles split of the continuous expression data, were also tested against DMFS. miR-106b was the only miRNA which had a significant area under the curve by ROC analysis in relation to DMFS. Furthermore Kaplan Meier curve analysis with miR-106b gave a significant separation between good and bad prognosis, -independent of the type of cut-off value chosen (ROC cut-off, median and tertiles). ROC-curve analysis showed that a threshold of >0.0172 expression (area under curve: 0.665), was optimal for identification of patients at risk of developing distant metastasis. This threshold was used to divide the patient in two groups (≤0.0172 = low expression of miR-106b (n = 75), >0.0172 = high expression of miR-106b, n = 128)). Kaplan-Meier survival analysis showed that 4 of 75 ( = 5%) patients with low expression of miR-106b developed distant metastasis, while 25 of 128 ( = 19%) patients with high expression. Survival rates were 95% vs. 81%, respectively (P = 0.004) (Figure 2A). In addition, miR-106b is significantly correlated with ERα negative, TNP and CK5/6 positivity.

Univariate cox-regression of all the clinicopathological parameters for DMFS showed that only tumour diameter, Nottingham grade, Her2, MAI, Ki67, PPH3 and miR-106b were significant. A multivariate survival analysis, including all features that were significant by univariate analysis, showed that Her2 and miR-106b were the strongest prognostic factors for DMFS (HR = 5.5, 95% CI 1.6–18.5). Her2 positive patients with low expression of miR-106b have a DMFS of 83% vs. 56% for patients with high expression (Table 3). miR-106b expression shows also a trend for additional prognostic information in the group of patients with high proliferation (MAI≥10) (Figure 2B), with respectively 100% vs. 69% (p = 0.063) for high vs. low expression of miR-106b.

Since some of the microRNAs correlated to the same clinicopathological parameters we used hierarchical cluster analysis to illustrate the expression level in all patients in comparison with the different parameters (Figure 3). Figure 3 is a supervised hierarchical cluster of ERα driven genes (Anova analysis, P<0.0001). This analysis confirms the tight connections between miR-18a and miR-18b to ERα negative cancers. Furthermore, high expression of let-7b was associated with favourable prognostic features (low proliferation, ERα/PR positivity, HER2/CK5/6 negativity and tumour size <2 cm) (cluster 1). As figure 3 indicates, a shift from high to low expression of let-7b points toward worse prognosis. Low expression of let-7b, miR-25 and miR-505 and little expression of miR-18a, miR-18b and miR-106b can identify a small group of patients (cluster 2) in which 8 of 25 patients develop distant metastasis. While patients with both high proliferation, TNP, CK5/6 positive, tumour size ≥2 cm and grade 3 cluster significantly together and have high expression of miR-505, miR-18a/b, miR-25, miR-106b and low expression of let-7b (Figure 3, cluster 3).

## Discussion

The current validation study by means of another analytical method and in FFPE material of new LN- breast cancer patients shows many of the same correlations between miRNAs and clinicopathological features as described before [15]. Moreover, miR-106b had strong prognostic univariate and multivariate prognostic value.

The family of miRNA let-7b was one of the first miRNAs to be discovered as non-coding RNA [25] and has been well investigated. Let-7b has been reported as a tumour-suppressor gene in cancers of the breast [26], pancreas [27] and stomach [28]. Some suggest that let-7b and its family regulate ERα activity and expression [29], [30], others show that let-7b expression correlates to luminal subtypes, thereby suggesting that tumours which maintain let-7b expression are less metastatic [26]. These findings support our current results that high expression of let-7b is associated with patients with favourable prognostic factors like low proliferation, ERα/PR positivity, Her2/CK5/6 negativity and tumour size <2 cm.

Our data also suggest that miR-29c acts as a tumour suppressor gene, as it shows low expression in TNP, ERα negative or CK5/6 positive cancers. In a recent, elaborate miRNA profiling study containing 207 breast cancers high expression of miR-29c was also associated with good prognosis [31]. Tumour necrosis factor alpha-induced protein 3 (TNFAIP3), a key regulator in inflammation and immunity, was found to be inversely correlated with miR-29c levels and was identified as a target of miR-29c [32]. Furthermore, high TNFAIP3 expression levels were observed in more aggressive breast tumours (ERα/PR negative and high histological grade) [33]. Other reports indicate the same in lung cancer [34] and nasopharyngeal carcinomas [35], while others state it as up-regulated in breast cancer tissue in comparison with normal adjacent tumour tissues [36].

Reports from both breast cancer tissue and breast cancer cell lines show that miR-18a and miR-18b are highly expressed in ERα negative breast cancer [37], [38]. Transfection of pre-miR-18a or pre-miR-18b to MCF7 cell lines, showed a reduced level of ERα mRNA and therefore it was concluded that ERα is a direct target for these miRNAs. Also in the same study, an increasing number of cells in G1/G0 phase of the cell cycle, when cells are stimulad with these miRNAs, was reported [38]. Previous results [15] and the current validation study show that high expression of miR-18a and miR-18b is associated with high proliferation, ERα negative, TNP and CK5/6 positivity, thereby indicating that these miRNAs could be markers for basal-like breast cancers. miR-18a and miR-18b have nearly identical sequences and our data show that their expression is strongly correlated. miR-106b is part of the same family as miR-18a and miR-18b, and has been reported up-regulated in several types of cancer, including colonic cancer [39], gastric cancer [40], laryngeal cancer [41] and breast cancer [42], [43]. Some of these findings suggest that miR-106b affects the cell cycle progression by targeting p21, a cyclin-dependent kinase inhibitor [42], [44]. Smith et al demonstrate a significant correlation between miR-106b and Six1 and activated TGFβ (nuclear Smad3), suggesting that high miR-106b leads to increased tumour initiating cell capacity and epithelial to mesenchymal transition [42]. Both these and our results indicate that miR-106b is a prognostic marker in breast cancer, but needs to be confirmed in independent study.

miR-106b is located in the intron of gene minichromosome maintenance protein 7 (Mcm7) [45], together with miR-25 and miR-93. Petrocca et al and others showed that BCL2L11 (Bim) is a direct target for miR-25 [46]–[48]. Bim protein is essential in regulation of apoptosis (review in [49]). Our results showed that miR-25 is significantly up-regulated in tumours with high proliferation, ERα negativity or CK5/6 positivity, all signs of low apoptosis. Although miR-106b and miR-25 are located in the same cluster and thereby thought to be expressed equally, we found no strong correlation between these miRNAs. This could signify a posttranscriptional mechanism that plays a key role in determining the levels of these mature miRNAs [50].

miR-505 is associated with apoptosis [51], [52]. Results of studies of drug-resistant breast cancer cell lines showed that transfection with miR-505 induced apoptosis by targeting alternative splicing factor/splicing factor 2 (ASF/SF2) [51]. Moreover, gene expression data from mouse mammary tumours associate miR-505 with basal-like breast cancer [53] This also agrees with our data that high expression of miR-505 correlates with positivity for CK5/6, ERα negativity and high proliferation.

There are only a few reports on miR-375 in breast cancer. Our data show that miR-375 is highly expressed in ERα positive and CK5/6 negative tumours. Down-regulation of miR-375 in colorectal cancer [54], squamous cervical cancer [55] and oesophageal cancer [56] suggests that miR-375 could target a tumour promoter gene. Newly published data showed that miR-375 expression was reduced in tamoxifen resistant cell lines compared with wild type cells [57]. That study observed an increased expression of epithelial markers, E-cadherin and ZO-1 mRNA level, and a decreased level of mesenchymal markers like (fibronectin, ZEB1and SNAI2/slug) after re-expression of miR-375. This suggests that miR-375 and one of it’s targets metadherin play a central role in the epithelial to mesenchymal (EMT) transition. Other publications that correlate miR-375 positivity with ERα expression in breast cell lines by targeting RAS dexamethasone-induced 1 [58] and the fact that higher level of miR-375 in has been detected in ERα negative breast cancer patient [59], fit well with this hypothesis.

As shown in figure 3 and 4 most of the miRNAs tested in this study are strongly related to proliferation, ERα and CK5/6; of these processes ERα is probably the most central. Although the correlation are strongly significant the effects can still be indirect, therefore we performed pathway analysis of the nine miRNAs by means of different software tools like Diana microT 4.0 (beta version), Pictar and Targetscan 5 [60]. The most significant (top three) pathway that is common in all three software programmes are mentioned and shown in table 4. Comparison of these pathways and the results presented in the current study clearly shows the complexity of the function of miRNAs as none of the analysis showed ER pathway, CK 5/6 or cell cycle as most important pathway.

On the other hand the fact that miRNAs can act on different processes at the same time is often used to suggest there potential as possible drug targets. Upregulation of let-7b and down regulation of miR-106b could be used for such strategy. This double action would in theory knock-out both the genomic and the non-genomic action of ERα and thereby possibly stop many of its carcinogenic roles.

The strength of this study is the fact that we validate the correlation between nine microRNAs and important clinicopathological features by a new method and in a new and larger cohort of patients. All patients come from the same hospital as the previous study, analyses of the clinicopathological features is all performed by highly standardized and reproducible methods.

The limitations of the study are the fact that total RNA was isolated from tissue with at least 50% tumour cells, so the expression levels might be different if pure tumour populations would be studied. In situ hybridization with the nine microRNAs could shed light on the location of the microRNAs in tissue and show whether the microRNAs are cell specific or not. Another important issue is the use of control molecules in qPCR experiments that involve microRNA. The use of U6 is strongly debated and different molecules have otherwise been used, in the current study we have analyzed all the data from our previous study by Normfinder and GeNorm in order to find the microRNA with the least variation in expressions levels.

In conclusion, we confirm our previously published data in a new and larger group of LN- breast cancer. These results indicate that high expression of miR-505, miR-18a, miR-18b, miR-25, miR-106b and low expression of let-7b are highly associated to unfavourable prognosis in LN- breast cancer patients. Furthermore, miR-106b has additional prognostic value to both MAI and Her2.

## Supporting Information

Figure S1 **Expression level of miRNAs vs MAI.** Independent T-test was used to determined significant relationship.
(TIF)

Figure S2 **Expression level of miRNAs vs ERα.** Independent T-test was used to determined significant relationship.
(TIF)

Figure S3 **Expression level of miRNAs vs PR.** Independent T-test was used to determined significant relationship.
(TIF)

Figure S4 **Expression level of miRNAs vs TNP.** Independent T-test was used to determined significant relationship.
(TIF)

Figure S5 **Expression level of miRNAs vs CK5/6.** Independent T-test was used to determined significant relationship.
(TIF)

Figure S6 **ROC-curve analyses of all miRNAs vs DMFS.**
(TIF)

Table S1 **ROC-curve analyses of microRNAs and the different biological features for breast cancer.**
(DOC)
