# Supplementary figures and images for: Validation of Expression Patterns for Nine miRNAs in 204 Lymph-Node Negative Breast Cancers
Source: PLoS One. 2012 Nov 7;7(11):e48692. doi: 10.1371/journal.pone.0048692 (PMC3492447; doi:10.1371/journal.pone.0048692)

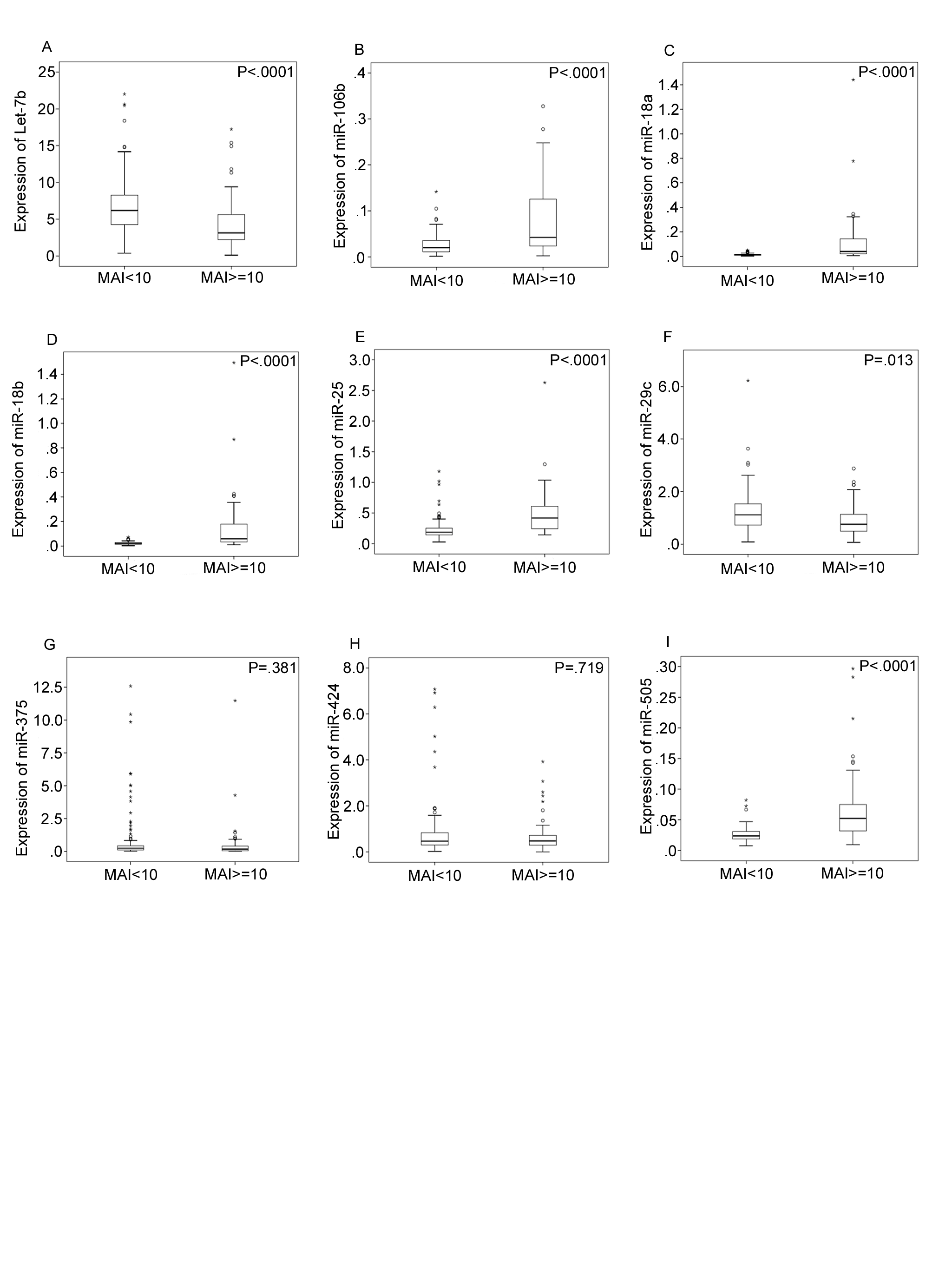

Supplement: Figure S1 — Expression level of miRNAs vs MAI. Independent T-test was used to determined significant relationship. (TIF) [file pone.0048692.s001.tif]

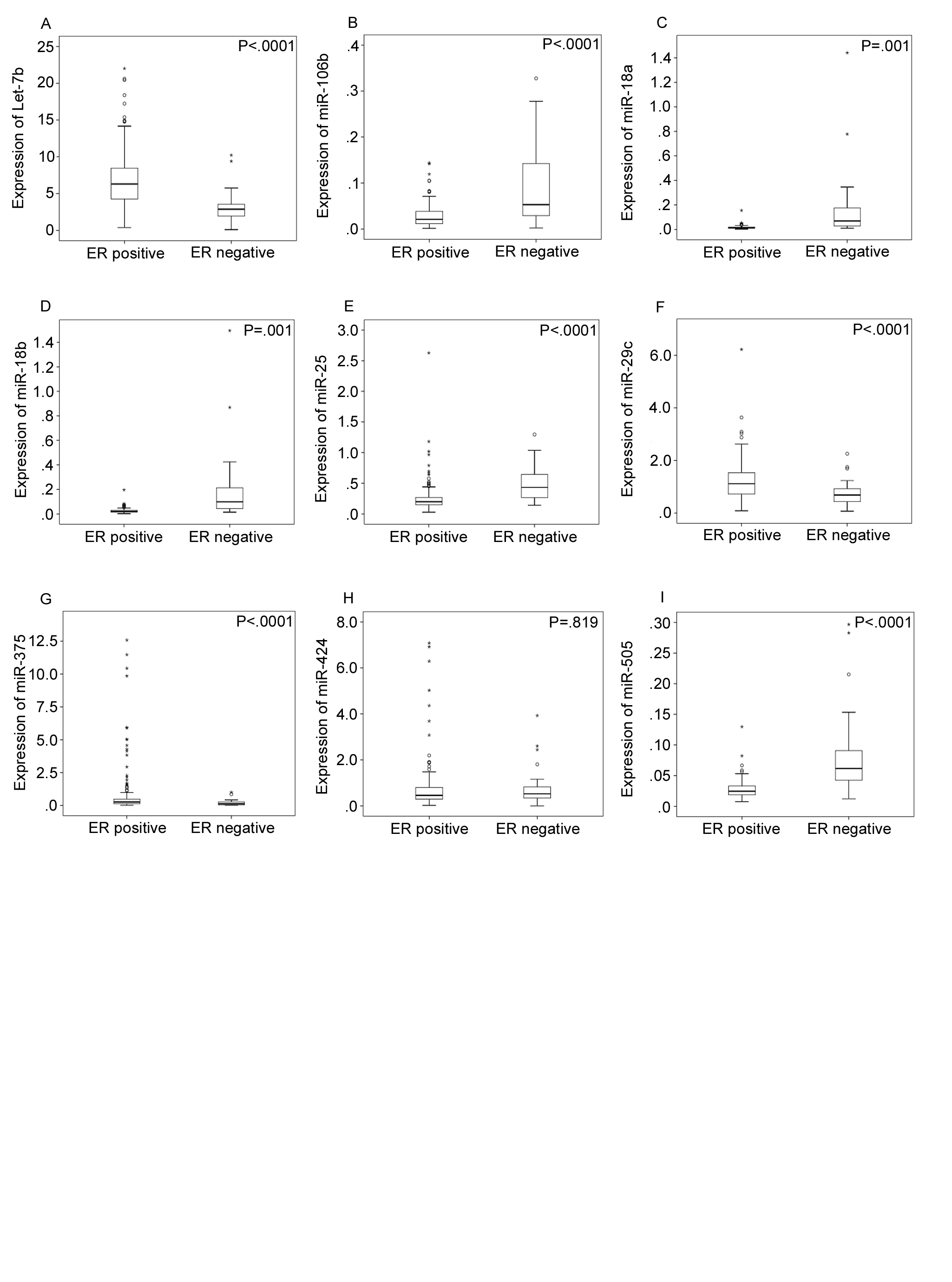

Supplement: Figure S2 — Expression level of miRNAs vs ERα. Independent T-test was used to determined significant relationship. (TIF) [file pone.0048692.s002.tif]

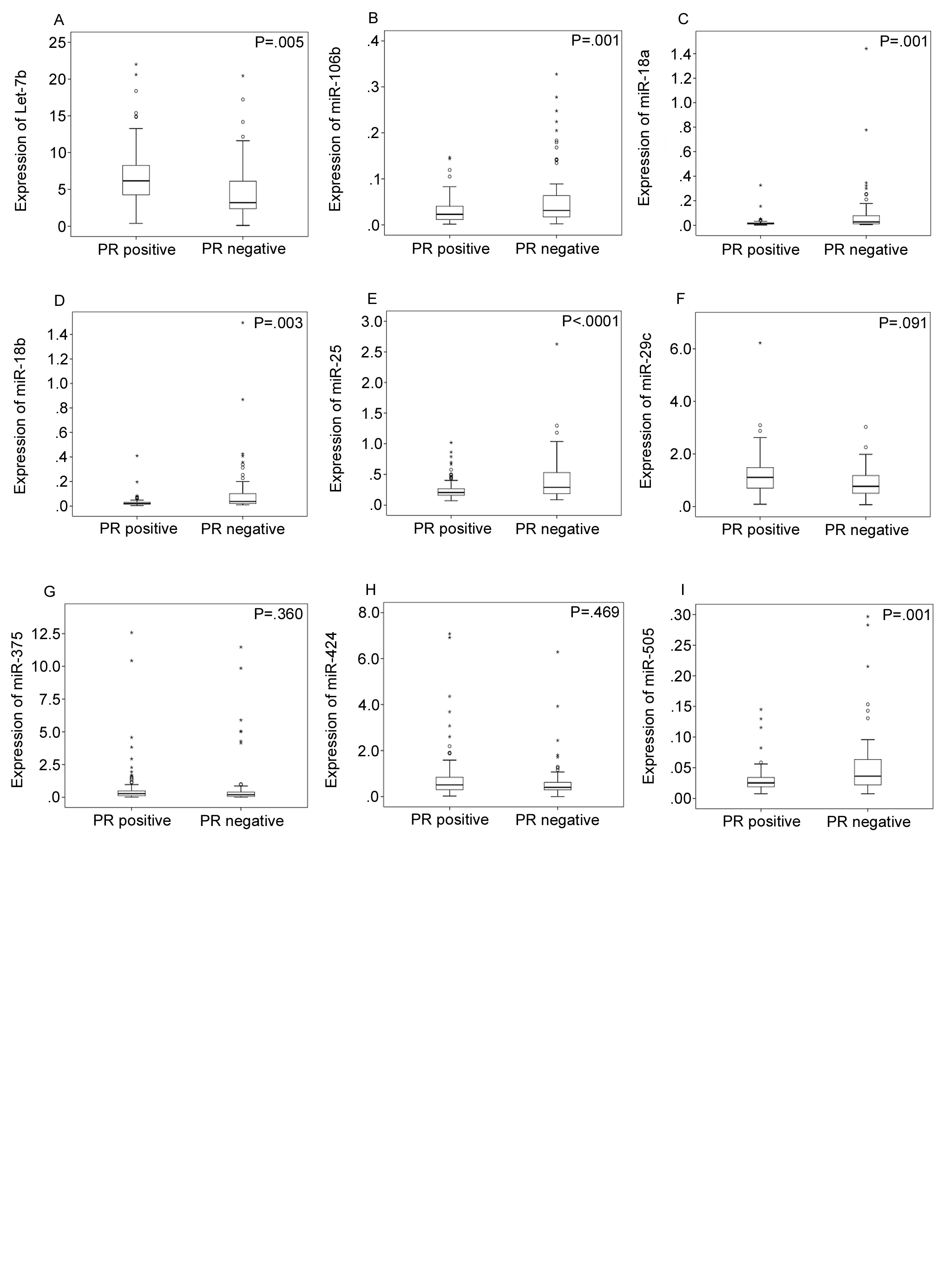

Supplement: Figure S3 — Expression level of miRNAs vs PR. Independent T-test was used to determined significant relationship. (TIF) [file pone.0048692.s003.tif]

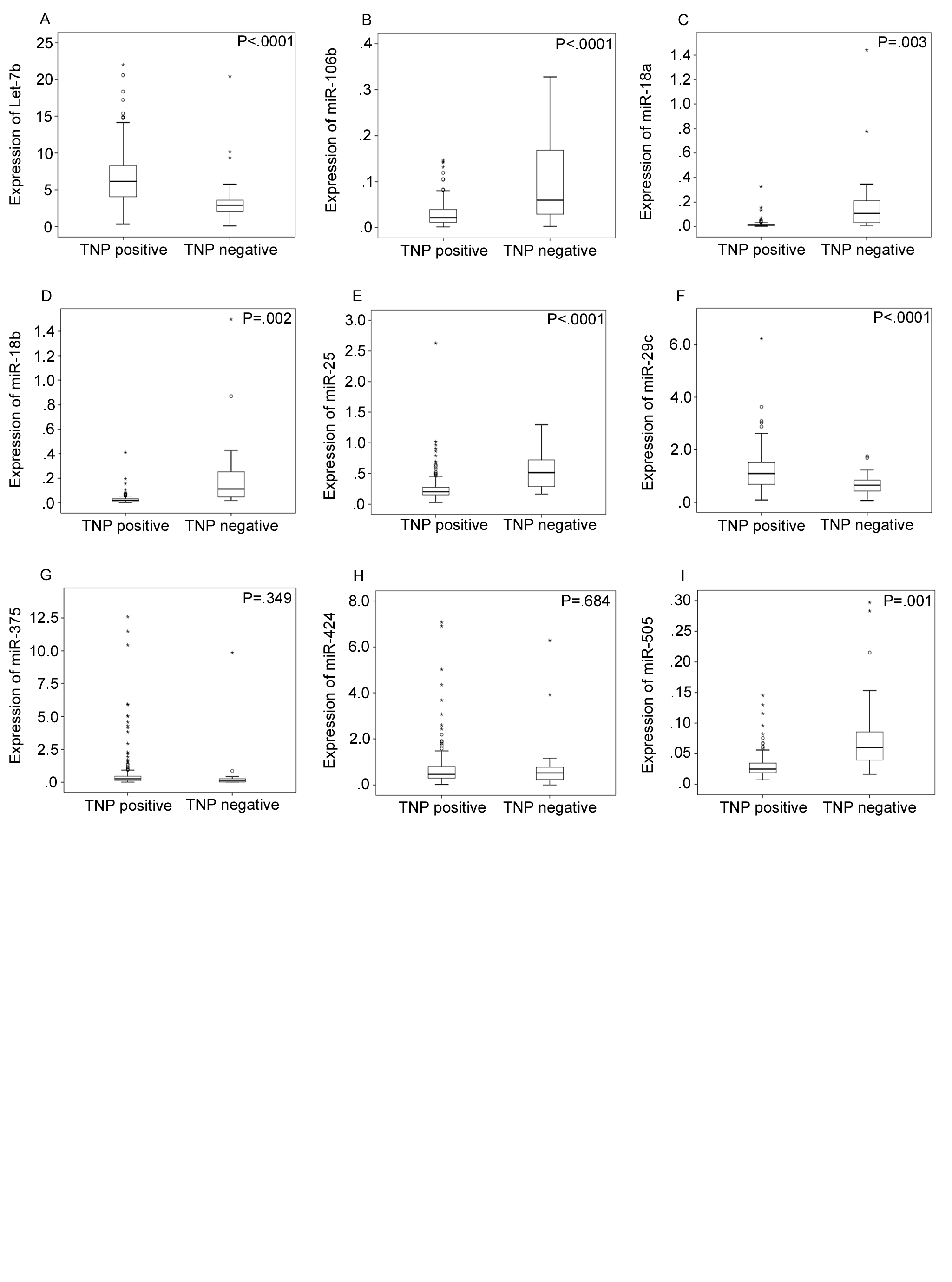

Supplement: Figure S4 — Expression level of miRNAs vs TNP. Independent T-test was used to determined significant relationship. (TIF) [file pone.0048692.s004.tif]

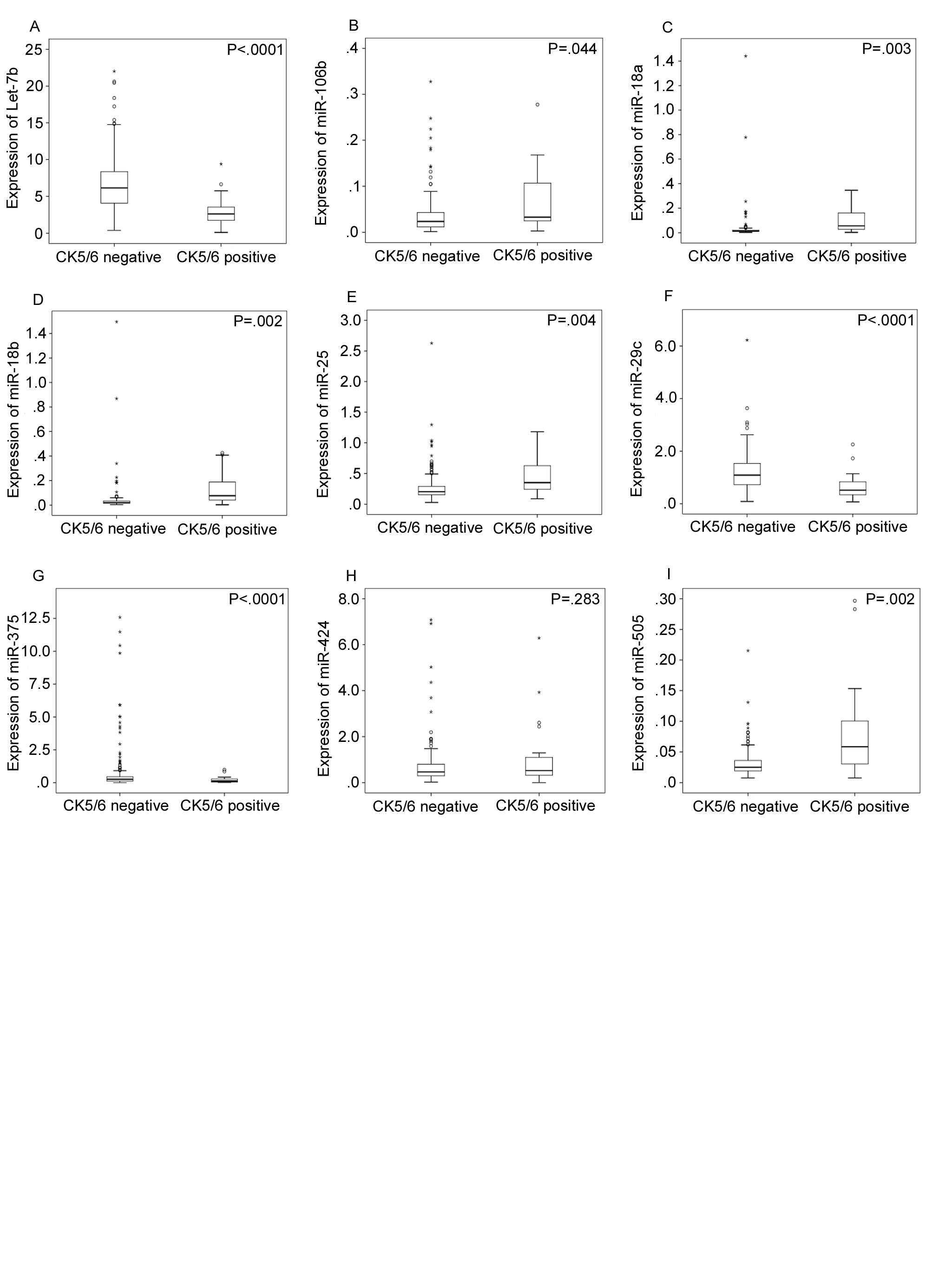

Supplement: Figure S5 — Expression level of miRNAs vs CK5/6. Independent T-test was used to determined significant relationship. (TIF) [file pone.0048692.s005.tif]

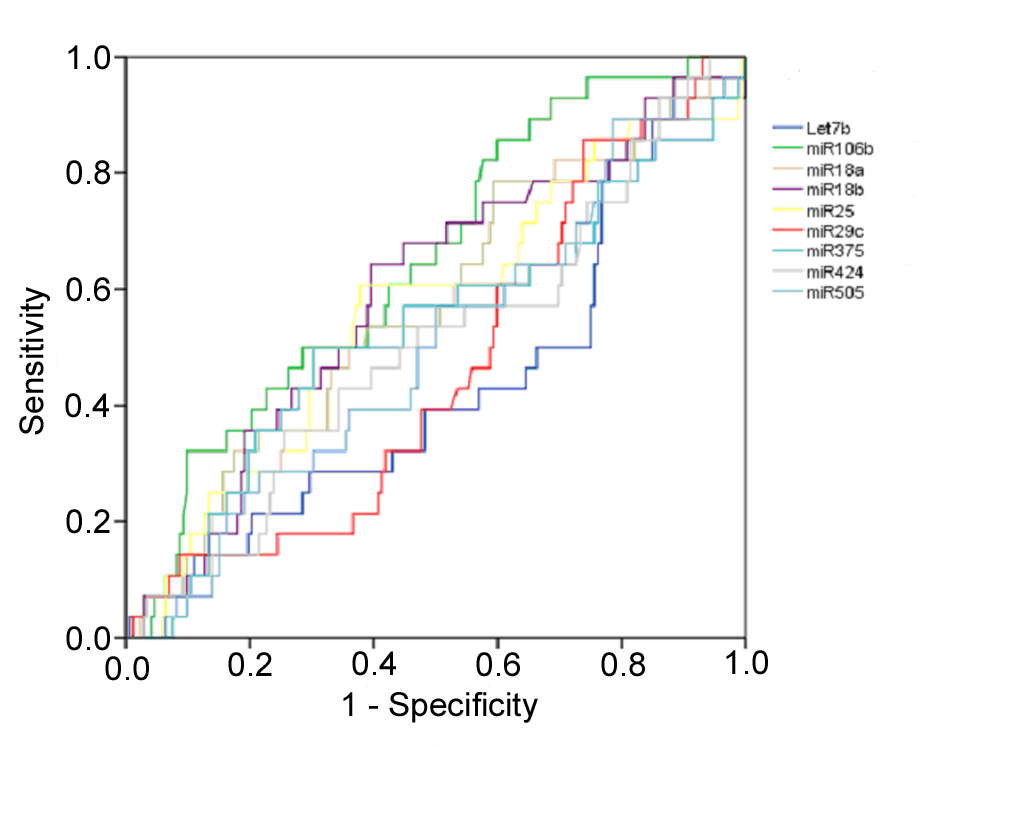

Supplement: Figure S6 — ROC-curve analyses of all miRNAs vs DMFS. (TIF) [file pone.0048692.s006.tif]
