# Supplementary material for: Validation of Expression Patterns for Nine miRNAs in 204 Lymph-Node Negative Breast Cancers
Source: PLoS One. 2012 Nov 7;7(11):e48692. doi: 10.1371/journal.pone.0048692 (PMC3492447; doi:10.1371/journal.pone.0048692)
Supplement: Table S1 — ROC-curve analyses of microRNAs and the different biological features for breast cancer. (DOC) [file pone.0048692.s007.doc]

**Table S1.** ROC-curve analyses of microRNAs and all the different biological features for breast cancer.

|  | Cut-off value | Area under curve | P-value (area =0.5) |
| --- | --- | --- | --- |
| **Let-7b** |  |  |  |
| DMFS | ≤ 3.2792 | 0.584 | 0.1233 |
| MAI | ≤ 3.1785 | 0.711 | 0.0001 |
| PPH3 | ≤ 3.4983 | 0.752 | 0.0001 |
| Ki67 | ≤ 5.5085 | 0.699 | 0.0001 |
| ERα | ≤ 4.8568 | 0.823 | 0.0001 |
| PR | ≤ 3.6008 | 0.643 | 0.0002 |
| Her2 | ≤ 5.3889 | 0.621 | 0.0298 |
| TNP | ≤ 3.6008 | 0.770 | 0.0001 |
| CK5/6 | ≤ 3.8018 | 0.821 | 0.0001 |
| **miR-106b** |  |  |  |
| DMFS | > 0.0172 | 0.646 | 0.0133 |
| MAI | > 0.0238 | 0.710 | 0.0001 |
| PPH3 | > 0.0238 | 0.723 | 0.0001 |
| Ki67 | > 0.0449 | 0.669 | 0.0001 |
| ERα | > 0.0711 | 0.728 | 0.0001 |
| PR | > 0.183 | 0.620 | 0.0030 |
| Her2 | > 0.042 | 0.628 | 0.0422 |
| TNP | > 0.0632 | 0.755 | 0.0001 |
| CK5/6 | > 0.0238 | 0.639 | 0.0200 |
| **miR-18a** |  |  |  |
| DMFS | > 0.0121 | 0.586 | 0.1502 |
| MAI | > 0.0196 | 0.867 | 0.0001 |
| PPH3 | > 0.0191 | 0.831 | 0.0001 |
| Ki67 | > 0.0134 | 0.833 | 0.0001 |
| ERα | > 0.0244 | 0.901 | 0.0001 |
| PR | > 0.0227 | 0.654 | 0.0001 |
| Her2 | > 0.0123 | 0.647 | 0.0191 |
| TNP | > 0.0227 | 0.899 | 0.0001 |
| CK5/6 | > 0.0266 | 0.822 | 0.0001 |
| **miR-18b** |  |  |  |
| DMFS | > 0.025 | 0.602 | 0.0860 |
| MAI | > 0.0289 | 0.860 | 0.0001 |
| PPH3 | > 0.0232 | 0.828 | 0.0001 |
| Ki67 | > 0.0232 | 0.829 | 0.0001 |
| ERα | > 0.0405 | 0.892 | 0.0001 |
| PR | > 0.0338 | 0.665 | 0.0001 |
| Her2 | > 0.022 | 0.659 | 0.0109 |
| TNP | > 0.0405 | 0.903 | 0.0001 |
| CK5/6 | > 0.0308 | 0.844 | 0.0001 |
| **miR-25** |  |  |  |
| DMFS | > 0.2509 | 0.560 | 0.3185 |
| MAI | > 0.2723 | 0.810 | 0.0001 |
| PPH3 | > 0.2529 | 0.769 | 0.0001 |
| Ki67 | > 0.2509 | 0.759 | 0.0001 |
| ERα | > 0.2529 | 0.794 | 0.0001 |
| PR | > 0.2529 | 0.639 | 0.0006 |
| Her2 | > 0.2529 | 0.630 | 0.0389 |
| TNP | > 0.2529 | 0.833 | 0.0001 |
| CK5/6 | > 0.2752 | 0.726 | 0.0001 |
| **miR-29c** |  |  |  |
| DMFS | ≤ 1.1674 | 0.522 | 0.6977 |
| MAI | ≤ 0.7605 | 0.632 | 0.0012 |
| PPH3 | ≤ 0.8971 | 0.612 | 0.0050 |
| Ki67 | ≤ 1.1674 | 0.585 | 0.0446 |
| ERα | ≤ 1.0023 | 0.718 | 0.0001 |
| PR | ≤ 0.7983 | 0.584 | 0.0359 |
| Her2 | ≤ 1.1878 | 0.524 | 0.6944 |
| TNP | ≤ 0.837 | 0.736 | 0.0001 |
| CK5/6 | ≤ 0.7038 | 0.764 | 0.0001 |
| **miR-375** |  |  |  |
| DMFS | > 0.3487 | 0.545 | 0.4490 |
| MAI | ≤ 0.1243 | 0.567 | 0.1188 |
| PPH3 | ≤ 0.1385 | 0.533 | 0.4233 |
| Ki67 | ≤ 0.1385 | 0.538 | 0.3730 |
| ERα | ≤ 0.1284 | 0.711 | 0.0001 |
| PR | ≤ 0.1284 | 0.568 | 0.0931 |
| Her2 | > 0.1241 | 0.590 | 0.1552 |
| TNP | ≤ 0.1284 | 0.738 | 0.0001 |
| CK5/6 | ≤ 0.1243 | 0.698 | 0.0001 |
| **miR-424** |  |  |  |
| DMFS | > 0.7702 | 0.518 | 0.7540 |
| MAI | ≤ 0.6674 | 0.514 | 0.7483 |
| PPH3 | > 0.4665 | 0.523 | 0.5754 |
| Ki67 | > 0.4303 | 0.550 | 0.2392 |
| ERα | > 0.3139 | 0.529 | 0.5743 |
| PR | ≤ 0.522 | 0.555 | 0.1760 |
| Her2 | > 0.2934 | 0.658 | 0.0114 |
| TNP | ≤ 0.2343 | 0.514 | 0.8085 |
| CK5/6 | > 1.0329 | 0.7558 | 0.518 |
| **miR-505** |  |  |  |
| DMFS | > 0.0182 | 0.513 | 0.8249 |
| MAI | > 0.0395 | 0.810 | 0.0001 |
| PPH3 | > 0.038 | 0.750 | 0.0001 |
| Ki67 | > 0.038 | 0.697 | 0.0001 |
| ERα | > 0.0395 | 0.868 | 0.0001 |
| PR | > 0.0426 | 0.642 | 0.0004 |
| Her2 | > 0.0361 | 0.658 | 0.0115 |
| TNP | > 0.0395 | 0.812 | 0.0001 |
| CK5/6 | > 0.0488 | 0.779 | 0.0001 |
